# Endoscopic resection of gastric subepithelial lesions: techniques and tips

**DOI:** 10.1016/j.vgie.2025.05.003

**Published:** 2025-06-02

**Authors:** Michael Lajin, Fateh Bazerbachi, Helen Sohn, Octavio Armas

**Affiliations:** 1 Sharp HealthCare, San Diego, California, USA; 2 CentraCare, St Cloud, Minnesota, USA

## Abstract

**Background and Aims:**

In the past, endoscopic resection of gastric subepithelial lesions (SELs) was restricted to small lesions confined to the submucosa. Larger and deeper lesions were resected surgically. Recent innovations in endoscopy have yielded multiple techniques for extending the boundaries and providing minimally invasive resection of larger and deeper lesions.

**Methods:**

This article presents the different endoscopic modalities for resecting gastric SELs. The video illustrates, through clinical examples, how to choose the appropriate resection method and provides technical tips for these techniques.

**Results:**

In this case series, we implemented different endoscopic resection modalities on the basis of endoscopic and endosonographic evaluation of the tumor and patient anatomical factors. Successful en bloc resection of gastric SELs was achieved in all patients without adverse events.

**Conclusions:**

Multiple techniques made endoscopic resection of larger and deeper gastric SELs feasible. Successful resection requires familiarity with these techniques' tips and tricks.

## Background and aims

In the past, endoscopic resection of gastric lesions was restricted to small mucosal and subepithelial lesions (SELs). Over the last decade, other techniques have emerged to enable en bloc resection of larger mucosal lesions^1^ and larger and deeper SELs.^2^, ^3^, ^4^, ^5^, ^6^, ^7^ This article presents a case series of 4 patients to illustrate the different endoscopic modalities for resecting gastric SELs. These techniques are (1) submucosal tunneling endoscopic resection (STER), which consists of entering a submucosal tunnel, dissecting the tumor attachments to the mucosa and the muscularis propria (MP), retrieving the tumor, and closing the mucosal entry. (2) Full-thickness resection using the gastric full-thickness resection device (FTR/gFTRD), which involves pulling the lesion inside a cap, deploying an integrated over-the-scope clip, and resecting it with an integrated snare. (3) Endoscopic submucosal dissection (ESD), which involves making a circumferential mucosal incision and dissecting the tumor attachments to the surrounding layers. (4) If the tumor has a deep extension to the MP/serosa, ESD might turn to an exposed endoscopic full-thickness resection (e-EFTR). This procedure might require decompressing the abdomen percutaneously in addition to closing the defect securely. (5) In select cases, laparoscopic endoscopic cooperative surgery (LECS) uses a combined endoscopic-laparoscopic approach.

Choosing the appropriate method depends on tumor characteristics such as size, texture, location, depth, and borders, and whether the tumor is endophytic or exophytic. Patient anatomical factors, such as a tight esophageal entry, can impede the introduction of resection/closure devices or the retrieval of the resected lesion.

## Patients and methods

Endoscopic resection of gastric SELs is shown in Video 1, available online at www.videogie.org.

### Patient 1

**Figure 1:**
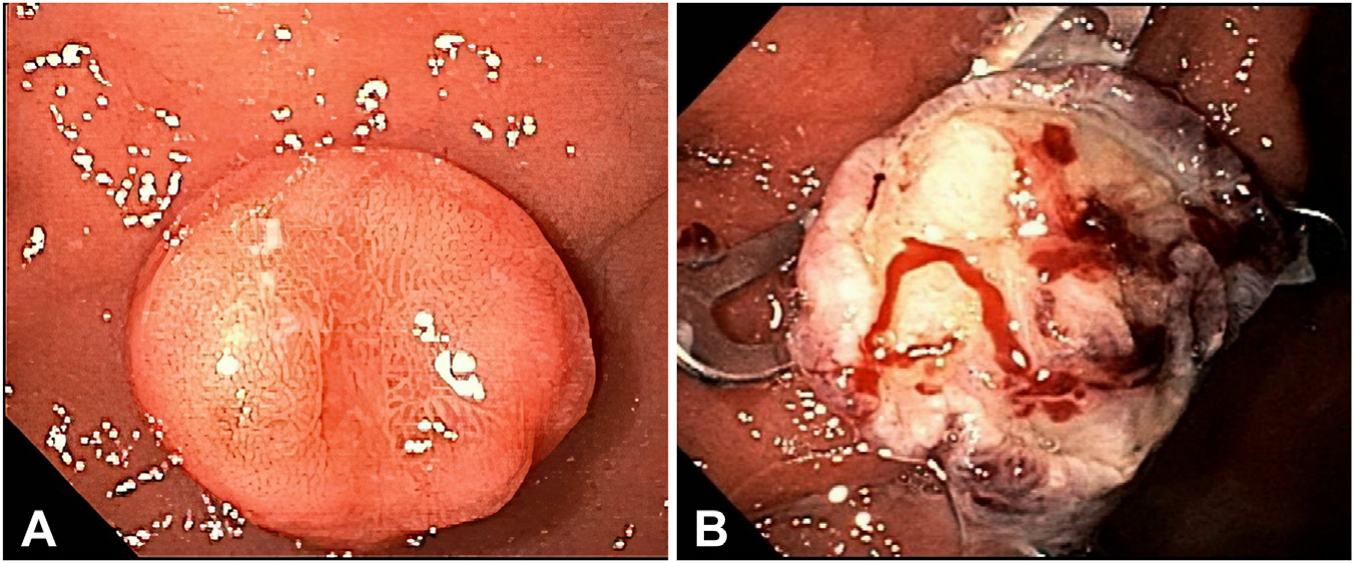
Patient 1. **A,** Endoscopic view of an umbilicated gastric subepithelial lesion. **B,** Endoscopic view after full-thickness resection using the gastric full-thickness resection device.

A 41-year-old man with a history of autoimmune metaplastic atrophic gastritis was found to have a gastric lesion with a nondiagnostic forceps biopsy. Upper endoscopy findings demonstrated a round, umbilicated SEL located at the gastric body, measuring 17 mm (Fig. 1A). EUS revealed an endophytic, mildly hypoechoic lesion involving the second and third layers. There was no lymphadenopathy or liver metastasis.

Given the endophytic orientation, the size of the lesion, and the suspected carcinoid, we elected to perform FTR using the gFTRD. Although ESD is a reasonable option, FTR/gFTRD is more efficient.

After marking the borders, the lesion was pulled inside the cap using an anchor. The over-the-scope clip was then deployed. The lesion was resected (Fig. 1B) and retrieved. Minor bleeding was treated with thermal coagulation.

The pathology confirmed a well-differentiated carcinoid invading the submucosa (SM) with free margins. The patient has been monitored over the last 4 years without recurrence or new lesions detected.

### Patient 2

**Figure 2:**
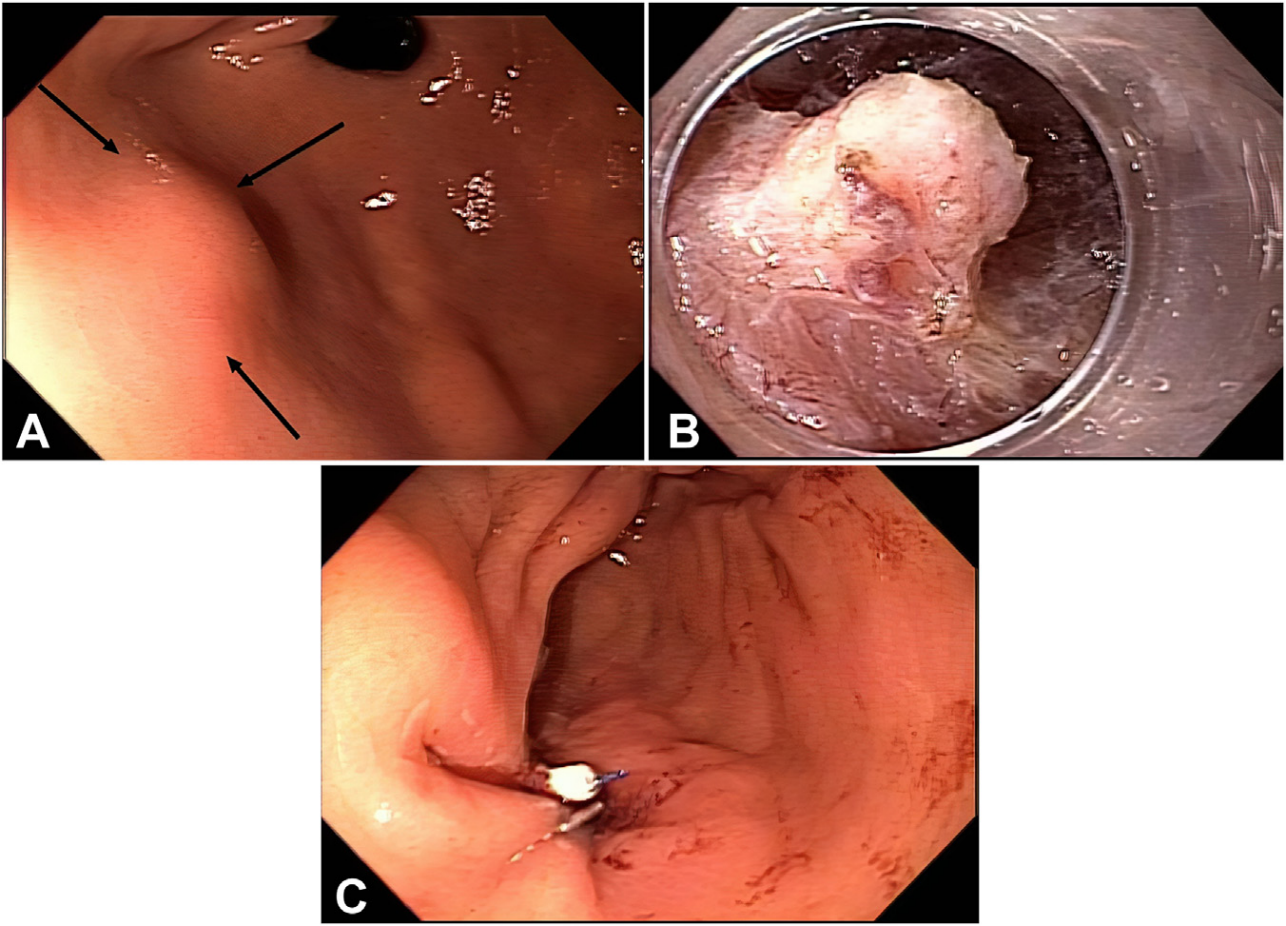
Patient 2. **A,** Endoscopic view of the gastric subepithelial lesion. **B,** Endoscopic view of the lesion during submucosal tunneling endoscopic resection after releasing the mucosal attachments. **C,** View after closing the mucosal tunnel entry with endoscopic suturing.

A 63-year-old woman was found to have a gastric SEL located at the anterior wall of the gastric antrum measuring 10 mm (Fig. 2A). EUS showed a hypoechoic lesion extending to the MP. The lesion was too small for a fine-needle biopsy, and the patient was noted to have a restricted hypopharyngeal space.

We discussed with the patient the options for surveillance versus endoscopic or surgical resection; she opted for endoscopic resection. We decided against gFTRD, given the tight esophageal entry. Although ESD and STER were considered appropriate for this lesion, we decided to proceed with STER because the location of this lesion provided easy access for tunneling. Compared with ESD, STER provides another layer of protection by keeping the mucosa overlying the tumor intact.

The borders of the lesion were marked. SM injection was performed 3 cm proximal to the lesion using a viscous solution. A horizontal mucosal incision was made using a HybridKnife (endocut; Erbe, Tübingen, Germany).

We have increasingly adopted the saline immersion therapeutic endoscopy (SITE) technique^8^, ^9^, ^10^ for our third-space procedures because of easier tunnel entry and safer dissection in narrow SM spaces. SM dissection using SITE (endocut) enabled entry into the SM tunnel.

The SM tunneling continued toward the lesion. Periodically, we came out of the tunnel to check our direction. Encountered blood vessels were coagulated with the same knife (preciseSECT; Erbe, Tübingen, Germany).

The SM tunneling was extended until the lesion was reached. The SM attachments at the tumor borders were dissected. The SITE technique was invaluable in navigating a narrow SM space and protecting the mucosa during the dissection of the SM attachments between the tumor and the mucosa.

After freeing the lesion from the SM attachments (Fig. 2B), we switched to carbon dioxide (CO_2_) insufflation when dissecting the tumor attachments to the MP for the following reasons: The SITE technique might result in a liquid spill into the peritoneum if the tumor had a deep extension into the MP.

In addition, we prefer a coagulation current with CO_2_ insufflation rather than the SITE technique with an endocut current during the dissection of the attachments to the MP to decrease the chance of bleeding at deeper levels.

The dissection plane of the tumor attachments to the MP targeted the muscular fibers at the fringes of the tumor. This strategy preserved the tumor's integrity and, at the same time, avoided a wide dissection, which might have resulted in an unnecessary full-thickness defect.

The dissection plane was accentuated by mobilizing the tumor in different directions using the distal attachment cap. The tumor was released intact after minimal superficial muscular dissection. The retrieved tumor measured 10 mm.

The tunnel entry was closed with endoscopic suturing (Fig. 2C). There were no adverse events after the procedure. The patient was discharged the next day. Pathology showed a benign elastofibroma; the patient did not require further endoscopic surveillance.

### Patient 3

A 72-year-old woman was found on endoscopy to have a gastric stromal tumor (GIST), which was confirmed by EUS-guided fine-needle biopsy. She was referred for endoscopic resection.

We discussed with the patient the different techniques for endoscopic resection and the alternative options, such as laparoscopic and LECS resections. After a multidisciplinary discussion with the patient and the surgical team, the decision was made to proceed with endoscopic resection. The surgical team is standing by if needed.

**Figure 3:**
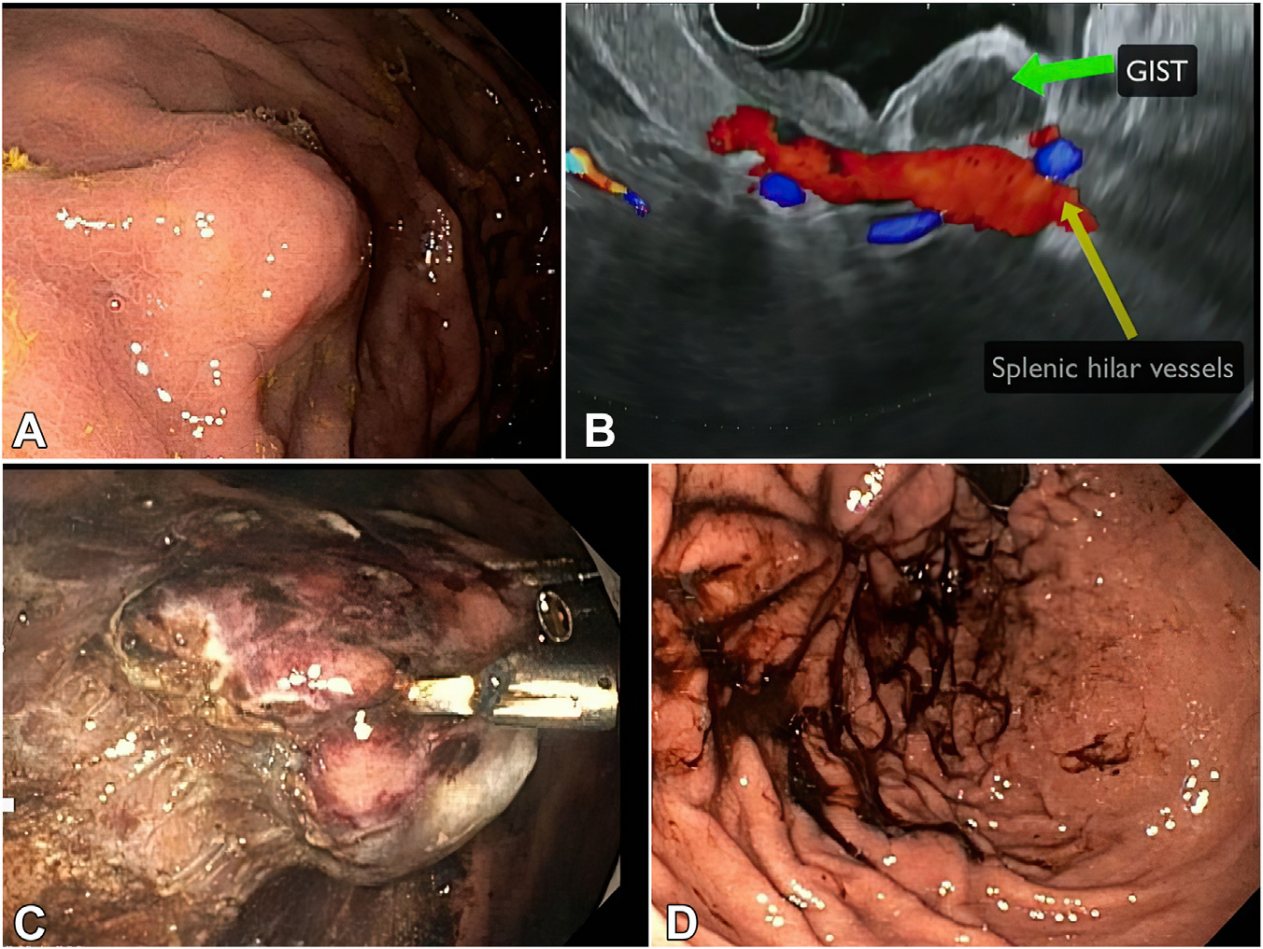
Patient 3. **A,** Endoscopic view of the gastric subepithelial lesion. **B,** Endosonographic view of the subepithelial lesion bordering the splenic hilar vessels. **C,** Traction to create tension on the tumor attachments and pull the tumor away from the splenic hilum. **D,** View after closing the full-thickness defect with endoscopic suturing.

Upper endoscopy revealed an SEL at the proximal greater curvature 4 cm below the esophageal-gastric junction (Fig. 3A). EUS showed a hypoechoic lesion measuring 2 cm, involving the MP, and bordering the splenic hilar vessels (Fig. 3B). After evaluating the different endoscopic resection modalities, we decided not to use the gFTRD, because the size and texture of the lesion could cause incomplete resection.

Entering a submucosal tunnel at the proximal greater curvature is challenging. As a result, STER was not a desirable option. In addition, STER does not allow traction, which is essential for the safe dissection of this lesion, given its proximity to the splenic hilar vessels. ESD and e-EFTR, in contrast, enable the use of traction to protect the blood vessels. As a result of the aforementioned reasons, we decided to proceed with ESD with a possible conversion to the e-EFTR technique if needed.

A prophylactic dose of antibiotics was administered before the procedure. The procedure was performed in the operating room with the patient under general anesthesia and in the supine position.

The borders of the tumor were marked using a coagulation current. SM injection with a viscous solution was followed by a circumferential mucosal incision with a HybridKnife (endocut mode). The SM fibers surrounding the tumor were dissected using the SITE technique (endocut mode).

At that point, traction was needed to create tension on the tumor attachments to the MP and to pull the tumor away from the splenic hilum. A clip attached to 1 circle of a figure 8-shaped string was deployed on the mucosa overlying the tumor. The other circle of the string was grasped with another clip and was deployed on the contralateral gastric wall. We switched to CO_2_ insufflation to distend the stomach and activate the traction.

The tumor attachments to the MP became under tension and were dissected carefully using an insulated-tip knife to avoid a deep organ or vascular injury (Fig. 3C). After the release of the muscular attachments, it became clear that the tumor was adhered to the serosa. Consequently, the procedure was converted to an e-EFTR. The serosal attachments were pulled back gently using an insulated scissor-type knife and dissected (coagulation current) under traction, exposing the peritoneum.

The abdomen was decompressed percutaneously using a 20-gauge catheter attached to a saline-filled syringe. As the dissection of the serosal attachments progressed, the traction became weaker because of insufficient insufflation of the gastric lumen. At that point, we decided to switch our traction to the helix-snaring technique^11^ to allow the safe release of the tumor from the remaining attachments.

The existing traction clip was released from the gastric wall. A 27-mm stiff snare was advanced through 1 channel, and a tissue helix was advanced through the other channel of a dual-channel endoscope. The snare was opened around the lesion. The helix was rotated and advanced inside the center of the lesion. The helix was pulled back, pulling the lesion away from the peritoneum. The tumor seemed to be attached to the gastric wall with a mucosal bridge. The snare was then closed slowly after ensuring the capture of the entire tumor. The snare cut the final attachments. The lesion was released entirely from the gastric wall en bloc and retrieved for pathological evaluation. The tumor measured 20 mm and had an intact capsule.

The resection site had a full-thickness defect with no bleeding. The full-thickness defect was closed with endoscopic suturing (Fig. 3D).

Given the proximity to the hilar vessels, we avoided taking suturing bites through the defect. Instead, full-thickness bites were taken through the gastric folds surrounding the defect.

The patient tolerated the procedure well without immediate adverse events. At 40 hours postprocedure, an upper gastrointestinal series confirmed good closure with no contrast leak. The diet was advanced, and she was discharged 2 days later on a short course of oral antibiotics. Pathology showed a spindle-cell type GIST. The tumor invaded the MP. The excisional margins were clear.

### Patient 4

**Figure 4:**
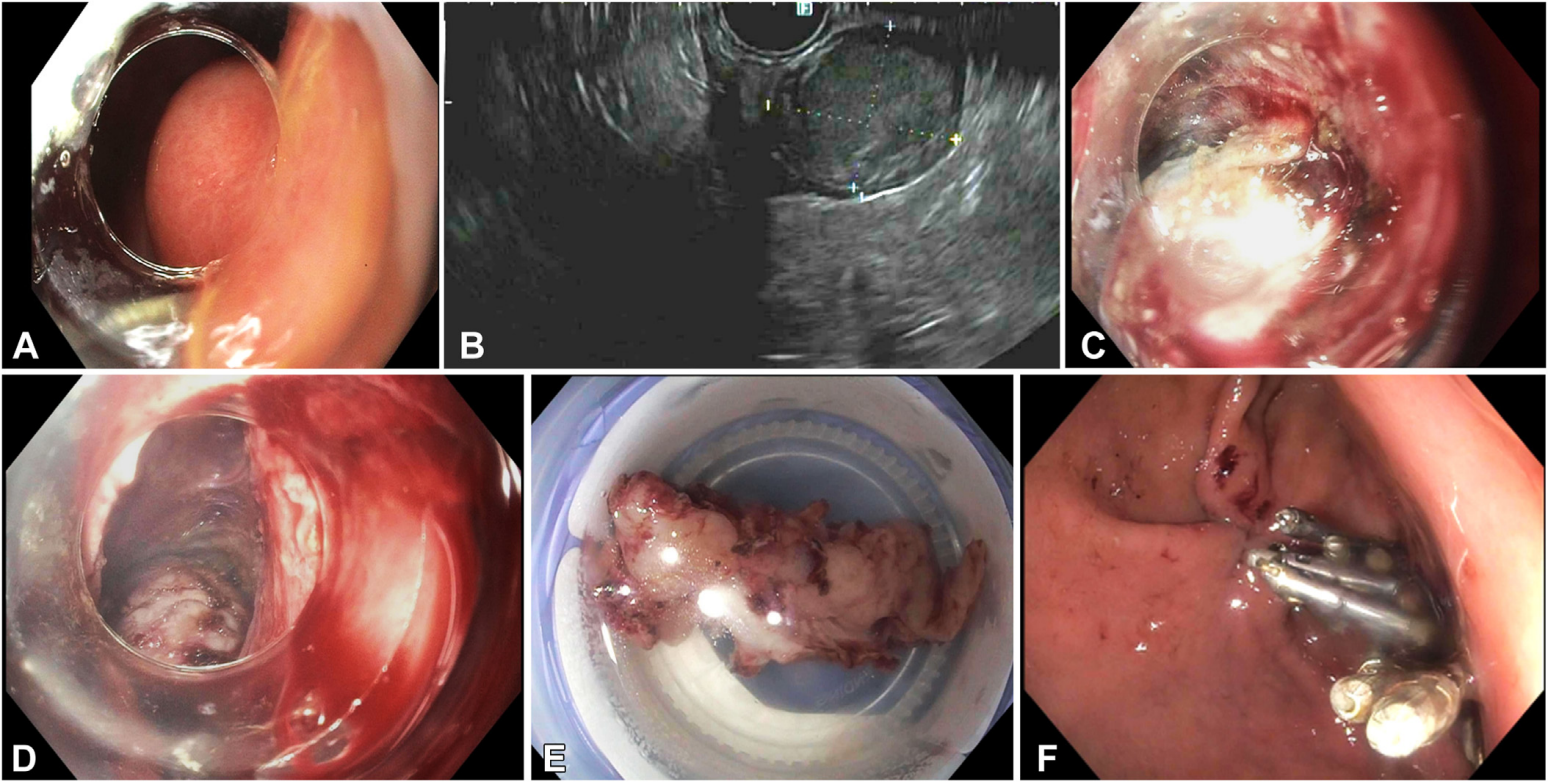
Patient 4. **A,** Endoscopic view of the gastric subepithelial lesion. **B,** Endosonographic view of the gastric SEL. **C,** Endoscopic view of the SEL after submucosal tunneling. **D,** Endoscopic view of the SEL after dissecting its attachments to the muscularis propria. **E,** The extracted gastric SEL measuring 5 cm. **F,** View after closing the tunnel entry with endoscopic suturing and clips. *SEL*, Subepithelial lesion.

A 58-year-old man was found to have a gastric SEL located at the lesser curvature of the distal stomach, pre-incisural, measuring approximately 2 cm on endoscopic views (Fig. 4A). EUS showed a hypoechoic lesion involving the MP with an estimated size of 2 cm (Fig. 4B). The lesion was confirmed as a GIST by EUS-guided fine-needle biopsy.

After evaluating the different treatment options, including surgical wedge resection, the case was discussed in a multidisciplinary fashion with the surgical team. The surgeons deemed a wedge gastrectomy high risk because of difficulty visualizing this area laparoscopically and the risk of vagal nerve injury. Therefore, we decided to proceed with STER as the initial approach, with the surgical team standing by if needed.

The procedure was performed in the operating room with the patient under general anesthesia with laparoscopic backup available. After marking the borders, SM injection was performed, and a horizontal mucosal incision was made. SM dissection using the SITE technique enabled entry into the SM tunnel.

As the dissection progressed (Fig. 4C), it became evident that the lesion was significantly larger than the estimated size by EUS (no computed tomography scanning was performed preoperatively). Moreover, the lesion had an exophytic component extending into the peritoneum.^12^

After complete dissection of the lesion from its attachments (Fig. 4D), it became clear that extraction through the SM tunnel would not be possible because of the lesion's size. In addition, oral retrieval was not feasible without the risk of injury to the patient or compromising the specimen's integrity.

At this point, the procedure was converted to an LECS approach.^13^ The laparoscopic team was called in, and the lesion was handed to the surgeon laparoscopically by carefully delivering it through the peritoneum via the endoscope using a rat-tooth forceps. The lesion was extracted intact via a laparoscopic port. It measured 5 cm (Fig. 4E).

The SM tunnel entry was closed with endoscopic suturing and reinforced with clips (Fig. 4F). The surgeon confirmed the lack of any occult perforation and adequacy of the closure via a leak test.

The patient had an uneventful recovery and was discharged 24 hours later. Pathology confirmed a spindle-cell GIST with clear margins.

This case illustrates the following teaching points:

1. Size underestimation of gastric SELs by EUS can occur, highlighting the importance of cross-sectional imaging in select cases.
2. Combined laparoscopic-endoscopic approaches can facilitate both the resection and extraction of large specimens without compromising oncologic principles or patient safety.

## Conclusions

A thorough endoscopic, endosonographic, and in select cases, cross-sectional imaging evaluation is a sine qua non for planning an endoscopic resection of a gastric SEL. Successful endoscopic resection of gastric SELs requires acquaintance with the tips and tricks of the different resection techniques. Safe endoscopic resection of gastric SELs requires proficiency in closure techniques and percutaneous abdominal decompression. It also requires a multidisciplinary approach and surgical support.

## Patient Consent

The patients in this article have given written informed consent to publication of the case details.

## Disclosure

All authors disclosed no financial relationships.

## Supplementary data

Video 1 Endoscopic resection of gastric subepithelial lesions: techniques and tips.
